# Severe Injury of the Abdomen, with Protrusion of the Intestines, Followed by Recovery

**Published:** 1844-03-09

**Authors:** Hattersly P. Worthington

**Affiliations:** Elkridge Landing, Md.


### THE MEDICAL EXAMINER,

### And Record of Medical Science.

Vol. VII.] PHILADELPHIA, SATURDAY, MARCH 9, 1844. [No. 5.

### SEVERE INJURY OF THE ABDOMEN, WITH PROTRUSION OF THE INTESTINES, FOLLOWED BY RECOVERY.

BY HATTERSLY P. WORTHINGTON, M. D.

To the Editor of the Medical Examiner.

SIR:—Having read in the number of your journal for January 27, a communication from Prof. Dunglison, with the observations of Dr. Blundell upon abdominal surgery, I am induced to send you the following sketch of a case which lately came under my observation, and which forcibly illustrates the truth of Dr. B.'s conclusions as to the peritoneum, under severe injury to the abdomen. I regret that my report must be extremely imperfect, as I write entirely from memory; yet as the case possesses much interest, I am unwilling to withhold its publication.

Nov. 8th, 1843, I was called to John Newcomb, labourer in the iron mines, ætat. 40, tall, robust, healthy, and of steady habits. He was lying in a shanty or cabin an eighth of a mile from the bank. The account given me was, that about thirty minutes before I saw him he was engaged in shovelling earth into a cart, when a portion of the perpendicular bank, beneath which he was working, caved in, falling upon his legs, and projecting his abdomen against the tail of the cart; the horse at the same time starting forward lessened the force of concussion. He was conveyed on a board to the cabin where I found him. Before removing his clothes I perceived a portion of the intestines and mesentery, comprising about 15 inches of the colon, and 30 of the small bowel, protruding from a wound in the right groin, and lying upon the upper part of the thigh. Raising this mass, I perceived a wound extending along the whole course of Poupart's ligament, and downwards an inch between the genital organs and thigh; the edges of this wound were clean and uncontused, as though cut with the scalpel: the intestines showing no marks of violence. This wound was evidently produced by the contraction of the abdominal muscles during the fall.

A little to the left and below the umbilicus, there was a smaller wound, from which a small fold of the intestine presented itself. The edges and neighbourhood of this gave evidence of direct violence from the cart. The patient complained of no pain or uneasiness excepting an oppression in the region of the stomach, which spontaneous vomiting relieved by evacuating his dinner; the pulse about 70, full and soft.

I proceeded to reduce the intestines, in which I succeeded with no difficulty; bringing the wound together by the quilled suture, making three stitches at the distance of two inches apart. The smaller wound I closed by a few adhesive strips. The dressing, which occupied considerable time, caused my patient but little suffering. I had given him 50 minims (drops?) tinct. opii, and his pulse was less frequent than when I commenced.

There appeared at the wound hæmorrhage internally, though he gave no evidence of its being considerable.

My dressings were necessarily imperfect, not having immediately at command all things requisite for this purpose. The miserable accommodations, or rather want of accommodations, and the exposed situation of the shanty, afforded my patient but a poor prospect for the essentials to his condition, even should he fortunately escape the dangers of the first few hours.

Having before me less dread of any peritoneal inflammation, than the debilitating effects of so large a suppurating wound, I wished to pursue a course of treatment which, whilst it should spare my patient's strength, should be adequate against any peritonitis that might occur. I concluded to refrain from any *violent prospective* anti-phlogistics, and taking advantage of his present condition, I determined to keep my patient under the sedative influence of opium and refrigerants. On the 10th there was a *slight tympanitis* and *tenderness*, with *increased thirst*, and harder pulse, but these symptoms yielded rapidly to fomentations to the abdomen. The bowels were now, for the first time, moved by enema.

On the 11th, the patient was easy, surface cool and moist, pulse 60, regular and full; he sleeps well and soundly during the night.

On the 12th, he was removed by my directions to the Baltimore Infirmary, a distance of 10 miles, where, under the care of the distinguished Professor of Surgery of the University of Maryland, he has advanced rapidly to recovery.

The sketch I have given will serve to illustrate the question under consideration, and will furnish another evidence in favour of Dr. Blundell's views. That an abdominal wound of 8 inches in length, beside the smaller one, under such unfavourable circumstances, should have given rise to inflammation at no time requiring the abstraction of a single ounce of blood, is certainly a paradox to many of our profession.

A determination of what is "the rule," and what "the exception in such cases," would tend to impart a most useful confidence in this most important department of surgery. I presume there are many other young surgeons who, with myself, have had melancholy cause to deplore their early acquired dread of this bugbear peritonitis; and have found, when too late, that they have opened their heavy battery of anti phlogistics against an imaginary foe.

*Elkridge Landing, Md.*, Feb. 18, 1844.

